# Identification of keratin 19‐positive cancer stem cells associating human hepatocellular carcinoma using CYFRA 21‐1

**DOI:** 10.1002/cam4.1211

**Published:** 2017-09-30

**Authors:** Takayuki Kawai, Kentaro Yasuchika, Takamichi Ishii, Hokahiro Katayama, Elena Yukie Yoshitoshi, Satoshi Ogiso, Takahito Minami, Yuya Miyauchi, Hidenobu Kojima, Ryoya Yamaoka, Sadahiko Kita, Katsutaro Yasuda, Naoya Sasaki, Ken Fukumitsu, Etsuro Hatano, Shinji Uemoto

**Affiliations:** ^1^ Department of Surgery Graduate School of Medicine Kyoto University Kyoto Japan; ^2^ Department of Hepatobiliary Surgery and Liver Transplantation Pitié‐Salpétrière Hospital University of Pierre and Marie Curie (UPMC) Paris France; ^3^ Center for iPS Cell Research and Application (CiRA) Kyoto University Kyoto Japan

**Keywords:** cancer stem cells, CYFRA 21‐1, hepatocellular carcinoma, keratin 19, transforming growth factor beta receptor 1 inhibitor

## Abstract

The current lack of an easily measurable surrogate marker of cancer stem cells (CSCs) prevents the clinical application of CSCs for hepatocellular carcinoma (HCC). We previously reported that keratin 19 (K19) is a novel HCC‐CSC marker associated with transforming growth factor beta (TGFβ)/Smad signaling, and that K19^+^ HCC‐CSCs could be a new therapeutic target of TGFβ receptor 1 inhibitor LY2157299. In this study, we examined whether K19^+^ HCC‐CSCs can be tracked using cytokeratin 19 fragment CYFRA 21‐1. In 147 HCC patients who underwent curative resection and evaluated K19 expression by immunohistochemistry, preoperative serum CYFRA 21‐1 levels were significantly higher in K19^+^ patients than in K19^−^ patients (*P *<* *0.01). Receiver operating characteristic analyses revealed that serum CYFRA 21‐1 was the statistically significant and the most sensitive predictor of tumor K19 expression among preoperative laboratory test values (*P *<* *0.001). In HCC cells encoding with a K19 promoter‐driven enhanced green fluorescent protein, fluorescence‐activated cell sorting (FACS)‐isolated K19^+^ cells displayed significantly higher levels of supernatant CYFRA 21‐1 than K19^−^ cells (*P *<* *0.01). Gain/loss of K19 function experiments confirmed that CYFRA 21‐1 levels were regulated by K19 function in HCC cells. Furthermore, CYFRA 21‐1 levels reflected the treatment efficacy of LY2157299 in K19^+^ cells. In conclusion, CYFRA 21‐1 can be used to predict K19 expression in HCC, and should thereby aid in the development of novel therapeutic strategies targeting K19^+^ HCC‐CSCs.

## Introduction

Hepatocellular carcinoma (HCC), accounting for most cases of liver cancer, is the second leading cause of cancer‐related death worldwide 1. Although various treatments and multimodal therapies for HCC have been developed, the prognosis for HCC patients is far from satisfactory. To develop new HCC treatments, the clinical application of cancer stem cells (CSCs) should be advanced. CSCs possess the ability to self‐renew and differentiate into heterogeneous progenies with high motility and proliferation rates 2, 3. These stem cell‐like features of CSCs contribute to rapid tumor growth, the resistance of tumors to chemotherapy/radiotherapy, and the epithelial–mesenchymal transition (EMT) 4, 5, 6.

In HCC, various cell surface molecules and transcription factors expressed during hepatic organogenesis have been identified as CSC markers 7, 8, 9, 10, 11, 12, 13. Independently, we previously reported that keratin 19 (K19), a hepatic progenitor cell marker, is a novel HCC‐CSC marker associated with EMT and transforming growth factor beta (TGFβ)/Smad signaling, and that K19^+^ HCC‐CSCs could present a new therapeutic target of TGFβ receptor 1 inhibition 14. However, an easily identifiable surrogate marker of K19^+^ HCC‐CSCs that can be evaluated in blood samples is still lacking, and this has prevented us from fully predicting patient outcomes or evaluating therapeutic efficacies in HCC patients.

On the other hand, the cytokeratin 19 fragment CYFRA 21‐1 is known to be a useful biomarker in non‐small cell lung cancer 15. Additionally, the clinical usefulness of CYFRA 21‐1 has been demonstrated in various malignancies, including esophageal cancer, breast cancer, and pancreatic cancer 16, 17, 18. In HCC, although it was reported that high concentrations of CYFRA 21‐1 were often detected in patients with large tumor or portal vein tumor thrombus 19, the significance of CYFRA 21‐1 in HCC has been mostly unclear. Moreover, the relationship between K19^+^ HCC‐CSCs and CYFRA 21‐1 has not yet been elucidated. Based on this information, we hypothesized that K19 expression can be predicted by CYFRA 21‐1 in human HCC.

The aims of this study were to demonstrate that K19^+^ CSCs can be tracked by CYFRA 21‐1 in HCC. For this purpose, the expression of K19 was investigated in 147 human HCC surgical tissues. Subsequently, we examined the efficacy of serum CYFRA 21‐1 levels and various preoperative clinical factors in detecting K19 expression in HCC. Furthermore, a transgene vector that expressed enhanced green fluorescent protein (EGFP) under the control of the human K19 promoter was transfected into three HCC cell lines to visualize K19^+^ HCC‐CSCs 14. Using K19^+^/K19^−^ cells isolated via fluorescence‐activated cell sorting (FACS), we examined whether the CYFRA 21‐1 levels of culture supernatants were regulated by K19 expression in HCC cells.

## Materials and Methods

### Patients

In this study, patients diagnosed with HCC by computed tomography (CT) and/or magnetic resonance imaging (MRI) and performed curative resection at Kyoto University Hospital from January 2007 to December 2011, were included. Of these, patients with previous transarterial chemoembolization (TACE) and/or radiofrequency ablation (RFA), no consent for the use of surgical tissues, no available preoperative serum, diagnosis of cholangiocellular carcinoma by pathological examination, and difficulties in pathological assessment resulting from tumor necrosis, were excluded. Tumor recurrence was tracked until the death of the patient or the end of the study (March 31, 2016). Written informed consent for the use of resected tissue samples was obtained from all patients included in this study in accordance with the Declaration of Helsinki, and this study was approved by the institutional review committee of our hospital (Approval number, R0877).

### Immunohistochemistry

Immunohistochemical analyses were performed as previously reported 20, 21, 22. Mouse anti‐human K19 (Dako, Glostrup, Denmark) was used at a 1:100 dilution. K19 expression levels were semi‐quantitatively assessed. Samples were considered positive for K19 when K19 expression was observed in more than 5% of the tumor cells examined. Each slide was evaluated by two independent investigators (T.K. and K.Y.) who were blinded to the patient outcome by anonymizing the samples prior to assessment.

### Chemiluminescent enzyme immunoassay

Blood samples of HCC patients were collected and stored at 4°C for 1 h. Serum was separated by centrifugation (10,000*g* for 10 min) at 4°C, and then stored at −80°C until analysis. Culture supernatants of HCC cells were collected and stored at −80°C until analysis. In the experiments using TGFβ receptor 1 inhibitor LY2157299, culture supernatants of HCC cells were collected after 24 h of incubation with 0.5 *μ*mol/L LY2157299 or DMSO control. CYFRA 21‐1 levels were quantified using LumipulsePresto^Ⓡ^ CYFRA 21‐1 Chemiluminescent enzyme immunoassay (CLEIA) kits (Fujirebio, Tokyo, Japan) and LumipulsePresto^Ⓡ^ II (Fujirebio) according to the manufacturer's protocols. Each sample was tested in duplicate, and the results were averaged to maintain reliability.

### Generation of transgenic HCC cell lines

Human HCC cell lines Huh7, HLF, and PLC/PRF/5 were purchased from the American Type Culture Collection (ATCC, Manassas, VA). All cells were authenticated by short tandem repeat (STR) profiling analysis before receipt and were propagated for less than 6 months after resuscitation. HCC cells were cultured at 37°C with 5% CO_2_ in Roswell Park Memorial Institute 1640 medium (RPMI‐1640; Invitrogen, Waltham, MA) supplemented with 10% fetal bovine serum (FBS; MP Biomedicals, Santa Ana, CA), and penicillin/streptomycin (Meiji Seika, Tokyo, Japan).

We generated a transgene plasmid vector that expressed EGFP under the control of the human K19 promoter as described previously 14, 22. Briefly, the plasmid vector pHCK‐2952, which was constructed by inserting the human K19 promoter sequence into the pGL3‐Basic (Promega, Madison, WI) vector, was kindly provided by Professor Shuichi Kaneko (Kanazawa University, Kanazawa, Japan) 23. The human K19 promoter region was isolated from pHCK‐2952 by restriction digestion with the *Xho*I and *Hind*III enzymes (Takara Bio, Otsu, Japan), and then ligated to the plasmid EGFP‐1 (pEGFP1; BD Biosciences, Franklin Lakes, NJ), which was linearized by digestion with *Xho*I and *Hind*III. Subsequently, the transgenic vector was transfected into HCC cells using Lipofectamine LTX reagent (Invitrogen), according to the manufacturer's protocol. Stably transfected cells were selected by cultivation in the presence of 200 μg/mL G418 (Sigma‐Aldrich, St Louis, MO) over 30 days. We confirmed proper transgene insertion by polymerase chain reaction (PCR) and immunocytochemical analyses, as described previously 14, 22.

### Flow cytometry and single‐cell culture analysis

Cultured cells were dissociated with 0.05% trypsin‐EDTA (Wako, Osaka, Japan) solution and then resuspended in 2% FBS‐PBS. Dead cells were eliminated using 7‐amino‐actinomycin D (Beckman Coulter, Brea, CA) staining. Single‐cell culture analysis was then performed as previously described 14, 24, 25. Individual isolated cells were sorted into 96‐well culture plates using a FACSAria device (BD Biosciences), and the wells were visualized by light microscopy 10–16 h after sorting to confirm that each well contained only one cell. After isolation of each clone, the cells were expanded and subjected to flow cytometry analysis.

### Reverse transcription‐PCR (RT‐PCR), quantitative PCR (qPCR), and qRT‐PCR

Total RNA was extracted with PureLink RNA mini kit (Invitrogen). Approximately, 1 *μ*g of total RNA was then reverse transcribed into cDNA with Revertra Ace (Toyobo, Osaka, Japan). Primers were generated for the following genes: K19, K19 open reading frame, and β‐actin (Table S2). RT‐PCR analysis was performed as previously described 26, 27. qPCR and qRT‐PCR assays were performed using SYBR‐green PCR Master Mix (Applied Biosystems, Waltham, MA) on the ABI 7500 system (Applied Biosystems). Each target was run in triplicate, and the expression levels were normalized to those of β‐actin.

### Knockdown and overexpression of K19

For K19 knockdown experiments, HCC cells were transfected with 10 nmol/L K19‐siRNA (#4427037‐s7998 or #4427037‐s7999; Invitrogen) or control‐siRNA (#4390843; Invitrogen) using Lipofectamine LTX reagent (Invitrogen), according to the manufacturer's protocol. K19 expression was significantly downregulated by both K19 siRNAs (Fig. S1A). As identical results were acquired with both siRNAs, results of K19‐siRNA (#4427037‐s7999) are shown as representative data.

For K19 overexpression experiments, the human K19 open reading frame was amplified by RT‐PCR and ligated to the CMV6‐AC plasmid (PS100020; OriGene, Rockville, MD) digested with Sgf1 and RsrII. HCC cells were then transfected with this K19 expression vector or with mock vector (PS100020, OriGene) using Lipofectamine LTX reagent (Invitrogen), according to the manufacturer's protocol. K19 expression was significantly upregulated by the K19 expression vector (Fig. S1B). For quantification of CYFRA 21‐1, culture supernatants of HCC cells were collected 72 h after transfection.

### Reagents

The TGFβ receptor 1 inhibitor LY2157299 was obtained from Axon Medchem (Groningen, Netherlands). The compound was dissolved in 100% dimethyl sulfoxide (DMSO; Sigma) and diluted with RPMI‐1640 to the desired concentration with a final DMSO concentration of <0.5%.

### Statistical analyses

Statistical analyses were performed using SPSS version 20.0 (SPSS Statistics, Inc., Chicago, IL) and GraphPad Prism software version 6.0 (GraphPad Software Inc., San Diego, CA). Data are presented as the mean ± SD of three or more independent experiments. Student's *t*‐tests, Mann–Whitney *U* tests, Fisher's exact tests, chi‐squared tests, and log‐rank tests were used for analyses of statistical significance.

Recurrence‐free survival (RFS) and overall survival (OS) after the operation were calculated using the Kaplan–Meier method and analyzed with the log‐rank test. Significant variables from univariate analyses were included in a multivariate analysis using a Cox regression model. We plotted receiver operating characteristic (ROC) curves for serum CYFRA 21‐1 levels and preoperative laboratory test values, and calculated the area under each ROC curve (AUC). The optimal cutoff values for serum CYFRA 21‐1 were calculated using the maximum sum of sensitivity and specificity, as well as the minimum distance to the top‐left corner of the ROC curve. Statistical significance was defined as *P *<* *0.05.

## Results

### HCC Patients

**Figure 1:**
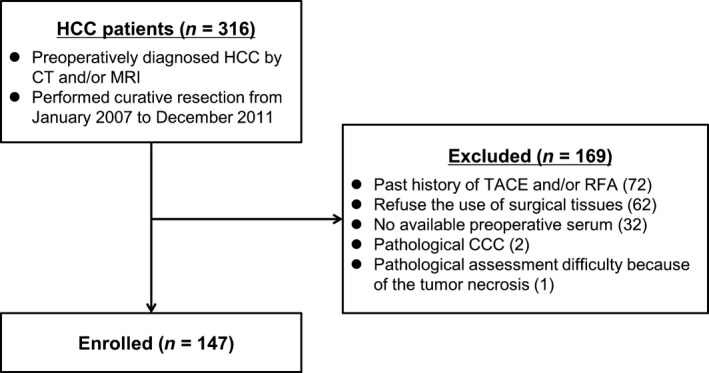
Flow of the patients through the study. HCC, hepatocellular carcinoma; CT, computed tomography; MRI, magnetic resonance imaging; TACE, transarterial chemoembolization; RFA, radiofrequency ablation; CCC, cholangiocellular carcinoma.

From January 2007 to December 2011, 316 consecutive patients diagnosed with HCC by CT and/or MRI were subjected to curative resection. Of these, 169 patients were excluded for the following reasons: previous TACE and/or RFA (72 patients), no consent for the use of surgical tissues (62 patients), no available preoperative serum (32 patients), diagnosis of cholangiocellular carcinoma by pathological examination (2 patients), and difficulties in pathological assessment resulting from tumor necrosis (1 patient). Thus, a total of 147 patients were included in this study (Fig. 1). The clinical pathological characteristics of the patients are summarized in Table S1. The follow‐up period from surgery until death or the endpoint of the study ranged from 79 to 3346 days (mean 1817 days).

### K19 expression in human HCC

**Figure 2:**
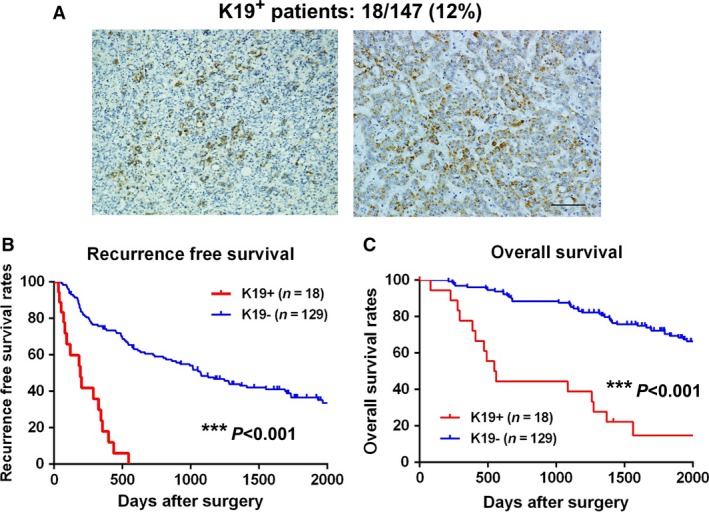
Keratin 19 (K19) expression in human hepatocellular carcinoma (HCC). (A) Representative images of K19^+^ HCC (left panel) and K19^−^ HCC (right panel). Scale bar represents 100 *μ*m. (B) Recurrence‐free survival rates according to K19 expression in the HCC tissues (log‐rank test, *** *P *<* *0.001). (C) Overall survival rates according to K19 expression in the HCC tissue (log‐rank test, *** *P *<* *0.001).

To detect K19 expression in human HCC clinical samples, 147 surgically resected primary HCC tumors were subjected to immunohistochemical analysis. K19 expression was observed in 18/147 (12%) cases (Fig. 2A). In K19^+^ HCC, K19 expression was detected in a large proportion or small proportion of HCC tissues (Fig. 2A). Some of K19^+^ cells were found in the invasive front of HCC. K19^+^ patients exhibited significantly reduced RFS and OS rates, with the median RFS being 193 days for K19^+^ patients and 1075 days for K19^−^ patients, and the median OS being 546 days for K19^+^ patients and 2893 days for K19^−^ patients (Fig. 2B−C and Table S3). The significance of K19 expression in predicting postoperative RFS and OS was confirmed by log‐rank test and multivariate analysis (Table 1 and Table S3). Specifically, K19 expression, serum CYFRA 21‐1 (≥ 2.7 ng/mL), tumor size (≥5 cm), the presence of multiple tumors, poor tumor differentiation, and microvascular invasion were associated with lower RFS rates according to log‐rank tests (Table S3). Log‐rank tests also revealed that K19 expression, serum CYFRA 21‐1 (≥2.7 ng/mL), high preoperative alpha‐fetoprotein (AFP) level (>20 ng/mL), tumor size (≥5 cm), the presence of multiple tumors, poor tumor differentiation, and microvascular invasion were associated with reduced OS rates (Table S3). Furthermore, multivariate analysis demonstrated that K19 expression had the highest hazard ratio among all independent predictors of both postoperative recurrence and reduced OS rates (Table 1).

**Table 1:** Multivariate analysis of factors predicting postoperative prognosis

Variables	Hazard ratio (95% CI)	*P*‐value
Postoperative recurrence		
K19 expression	5.81 (2.96–11.4)	<0.001
Tumor number (multiple)	1.72 (1.07–2.75)	0.024
Microvascular invasion	1.40 (0.88–2.22)	0.162
Tumor size (≥5 cm)	1.23 (0.81–1.97)	0.306
Poorly differentiated	1.24 (0.69–2.23)	0.471
CYFRA 21‐1 (≥2.7 ng/mL)	0.68 (0.28–1.63)	0.384
Postoperative survival		
K19 expression	4.79 (2.32–9.87)	<0.001
Tumor number (multiple)	1.74 (1.02–2.98)	0.043
Poorly differentiated	1.83 (0.94–3.59)	0.077
Tumor size (≥5 cm)	1.48 (0.86–2.54)	0.157
AFP (>20 ng/mL)	1.29 (0.73–2.29)	0.377
Microvascular invasion	0.98 (0.55–1.75)	0.949
CYFRA 21‐1 (≥2.7 ng/mL)	0.61 (0.22–1.69)	0.344

K19, keratin 19; CYFRA 21‐1, cytokeratin 19 fragment; AFP, alpha‐fetoprotein; CI, confidence interval.

### Efficacies of serum CYFRA 21‐1 level and preoperative laboratory test values predicting K19 expression in HCC

**Figure 3:**
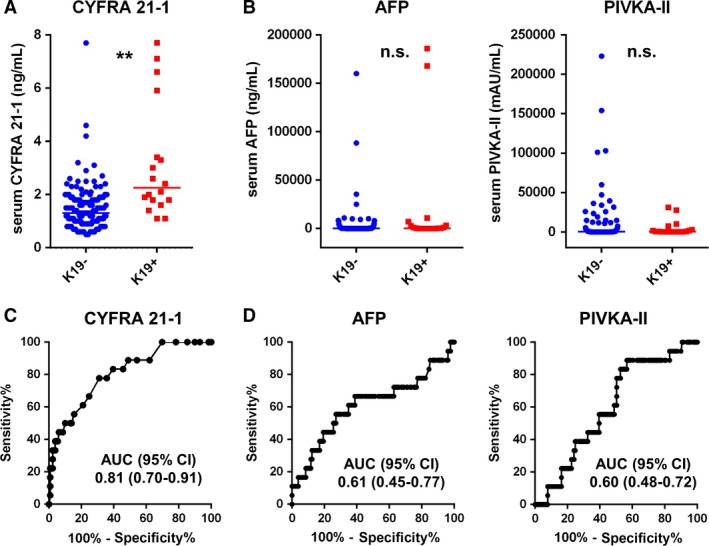
Relationship between K19 expression and serum cytokeratin 19 fragment (CYFRA 21‐1) level in human HCC. (A) Preoperative serum levels of CYFRA 21‐1 in K19/K19‐ HCC patients. K19^+^ patients had significantly higher serum CYFRA 21‐1 levels than K19^−^ patients (Mann–Whitney *U* test, **P <* *0.01). Each line indicates median level. (B) Preoperative serum levels of alpha‐fetoprotein (AFP) and protein induced by vitamin K absence 2 (PIVKA‐II) in K19/K19‐ HCC patients (Mann–Whitney *U* test, n.s.; not significant). Each line indicates median level. (C) Receiver operating characteristic (ROC) curve evaluating the performance of serum CYFRA 21‐1 level for predicting K19 expression in HCC. (D) ROC curve evaluating the performance of preoperative serum levels of AFP and PIVKA‐II for predicting K19 expression in HCC.

To determine the efficacy of serum CYFRA 21‐1 level in predicting K19 expression in HCC, we assessed the preoperative serum CYFRA 21‐1 levels of 147 patients. Preoperative serum CYFRA 21‐1 levels were significantly higher in K19^+^ (median = 2.3 ± 2.1) patients than in K19^−^ patients (median = 1.3 ± 0.9; *P *<* *0.01) (Fig. 3A). However, no significant differences were observed between K19^+^ and K19^−^ patients in terms of commonly used tumor markers for HCC, preoperative AFP levels, or the amount of protein induced by vitamin K antagonist‐II (PIVKA‐II; Fig. 3B). Notably, ROC analysis revealed that among the preoperative laboratory test values including AFP and PIVKA‐II, serum CYFRA 21‐1 level was the most sensitive predictor of K19 expression in HCC tumors (Fig. 3C−D and Table 2). Our ROC analysis also showed that the cutoff value of serum CYFRA 21‐1 level for the prediction of K19 expression was 1.8 ng/mL (sensitivity; 78%, specificity; 70%).

**Table 2:** Efficacy of CYFRA 21‐1 and preoperative laboratory test values for the evaluation of K19 expression in HCC

Factors	AUC (95% CI)	*P*‐value
CYFRA 21‐1	0.81 (0.70–0.91)	<0.001
AFP	0.61 (0.45–0.77)	0.130
PIVKA‐II	0.60 (0.48–0.72)	0.155
CA 19‐9	0.60 (0.45–0.74)	0.215
T‐bilirubin	0.59 (0.44–0.73)	0.242
CEA	0.57 (0.44–0.71)	0.336
Platelet	0.53 (0.37–0.70)	0.349
Albumin	0.44 (0.29–0.58)	0.392

AUC, area under curve; K19, keratin 19; HCC, hepatocellular carcinoma; CYFRA 21‐1, cytokeratin 19 fragment 21‐1; AFP, alpha‐fetoprotein; PIVKA‐II, protein induced by vitamin K absence 2; CA 19‐9, carbohydrate antigen 19‐9; CEA, carcinoembryonic antigen; CI, confidence interval.

### EGFP labeling of K19^+^ cell populations in heterogeneous HCC cell lines

To investigate the functional association between K19 and CYFRA 21‐1, we generated transgenic HCC cell lines to visualize K19^+^ cells and then analyzed K19^+^ and K19^−^ populations as previously described 14, 22. RT‐PCR analyses showed that the Huh7 and PLC/PRF/5 cell lines expressed K19, whereas HLF cells did not (Fig. S2A). Histological analyses demonstrated that Huh7 and PLC/PRF/5 cells contained both K19‐expressing and nonexpressing cells. Subsequently, each of these three cell lines was transfected with the K19‐EGFP reporter vector, and double staining of K19 and GFP confirmed that our selective GFP‐labeling method resulted in successful (efficiency > 95%) marking of K19‐expressing cells (Fig. S2B−D). Additionally, qPCR analyses confirmed that FACS‐sorted EGFP^−^ and EGFP^+^ cells contained equal copy numbers of the reporter gene (Fig. S2E−F). Individual FACS‐isolated K19^+^ cells generated both K19^+^ and K19^−^ cell fractions during single‐cell culture. In contrast, individual K19^−^ cells produced only K19^−^ cell fractions (Fig. S2G). Therefore, we further analyzed the K19^+^ and K19^−^ populations derived from a single K19^+^ cell.

### CYFRA 21‐1 levels in the culture supernatants of K19^+^ and K19^−^ HCC cells

**Figure 4:**
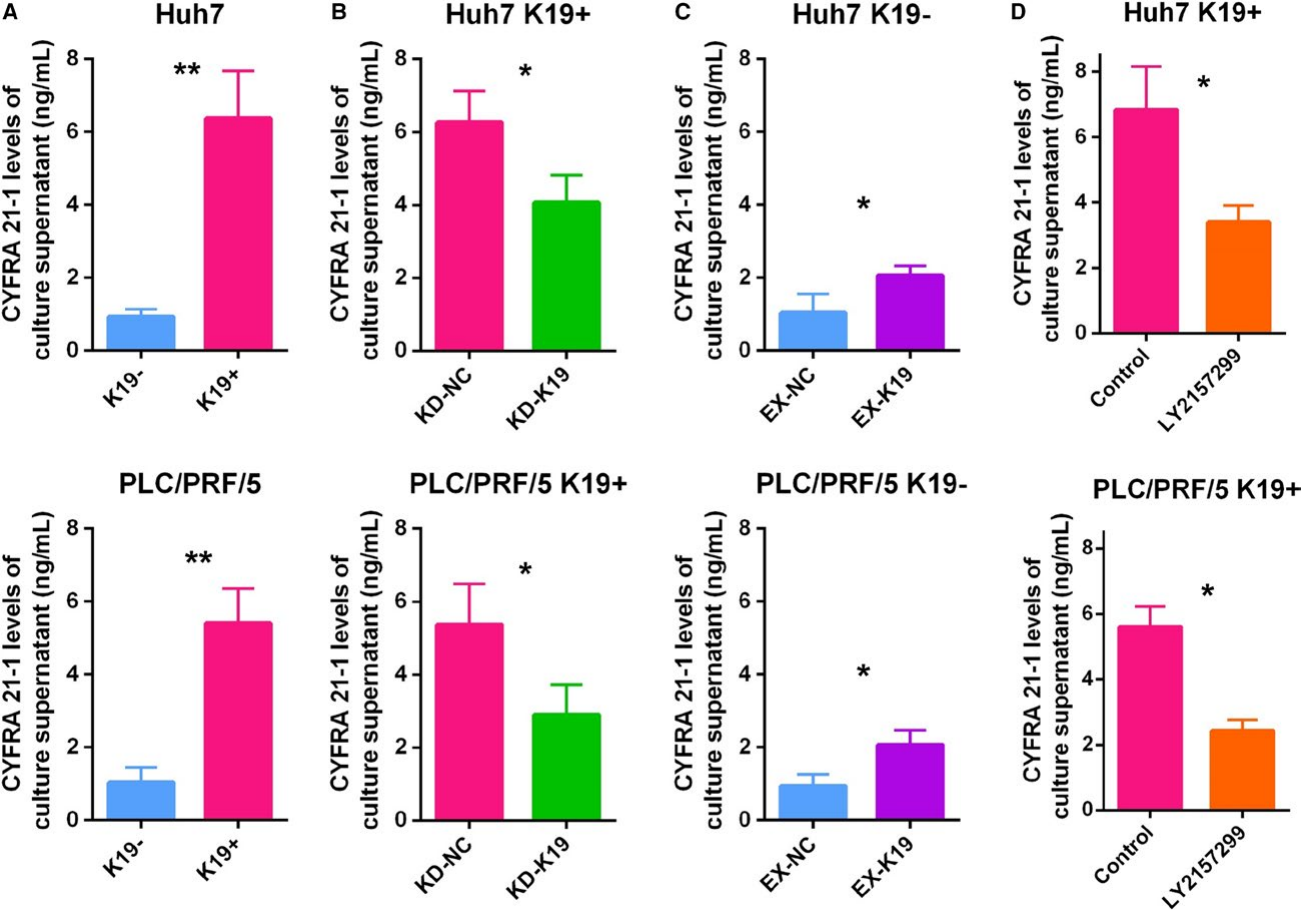
CYFRA 21‐1 level of the culture supernatants in K19^+^ and K19^−^ HCC cells. (A) K19^+^ cells showed significantly higher CYFRA 21‐1 levels of culture supernatants than K19^−^ cells (Student's *t*‐test, Huh7, **P <* *0.01; PLC/PRF/5, ** *P *<* *0.01). (B) CYFRA 21‐1 levels of culture supernatants were significantly suppressed by K19 knockdown in K19^+^ cells (KD‐NC, steady state K19^+^ cells; KD‐K19, K19 knockdown K19^+^ cells by K19‐siRNA) (Student's *t*‐test, Huh7, *P <* *0.05; PLC/PRF/5, *P <* *0.05). (C) CYFRA 21‐1 levels of culture supernatants were significantly elevated by K19 overexpression in K19^−^ cells (EX‐NC, steady state K19^−^ cells; EX‐K19, K19 overexpression K19^−^ cells) (Student's *t*‐test, Huh7, Huh7, *P <* *0.05; PLC/PRF/5, *P <* *0.05). (D) TGFbR1 inhibitor LY2157299 significantly suppressed CYFRA 21‐1 levels of culture supernatants in K19^+^ cells. (Student's *t*‐test, Huh7, *P <* *0.05; PLC/PRF/5, *P <* *0.05). Data are shown as the mean ± SD.

To determine the functional correlation between K19 expression and CYFRA 21‐1 levels in HCC, we first examined CYFRA 21‐1 levels in the culture supernatants of K19^+^ and K19^−^ HCC cells. CYFRA 21‐1 levels were significantly higher in K19^+^ Huh7 cells than in K19^−^ Huh7 cells (Fig. 4A). Subsequently, our gain/loss of K19 function experiments resulted in siRNA‐based K19 knockdown in K19^+^ Huh7 cells significantly decreased the CYFRA 21‐1 levels of the culture supernatants (Fig. 4B). In contrast, K19 overexpression in K19^−^ Huh7 cells consistently and significantly increased the CYFRA 21‐1 levels of the culture supernatants (Fig. 4C). Moreover, the treatment with LY2157299 resulted in significant suppression of CYFRA levels in K19^+^ Huh7 cells (Fig. 4D). Similar results were obtained in PLC/PRF/5 cells (Fig. 4A−D). These findings strongly suggest that CYFRA levels were regulated by K19 function in HCC cells, and that evaluation of CYFRA levels might therefore be useful for monitoring the response of K19^+^ HCCs to TGFβ receptor 1 inhibitors.

## Discussion

Recent developments in stem cell biology have revealed the existence of CSCs, which are associated with tumor recurrence/metastasis, in various cancers including HCC. HCC malignancies have one of the poorest prognoses due to the difficulty of controlling tumor recurrence/metastasis control. Therefore, the identification and clinical application of CSCs in HCC have attracted increasing attention. Although various HCC‐CSC markers have been reported, the clinical application of HCC‐CSCs has yet to be achieved. Independently, we previously reported that K19^+^ HCC‐CSCs with constitutive activation of TGFβ/Smad signaling could be treated with a TGFβ receptor 1 inhibitor, LY2157299. Consistent with previous reports 28, 29, 30, K19 expression was observed in approximately 15% of HCCs and was found to be an independent prognosticator of poor postoperative HCC outcomes in this study. Additionally, a separate study reported that K19 expression plays a key role in HCC invasion 31. For stratification of patients with highly malignant K19^+^ HCCs and development of clinical applications of K19^+^ HCC‐CSCs, it is necessary to identify an easily measurable surrogate biomarker of K19^+^ HCC‐CSCs.

In the field of clinical cancer treatment, CYFRA 21‐1 has been established as a useful biomarker for detection of cancer, prediction of tumor recurrence, and assessment of the therapeutic efficacy of chemotherapies in many cancers. However, the significance of CYFRA 21‐1 in HCC has been mostly unrevealed. Since many HCC patients are K19‐negative, CYFRA 21‐1, which is a fragment of K19, may be not useful in the diagnosis of HCC. However, considering the stability of CYFRA 21‐1, we hypothesized that K19 expression could be predicted by CYFRA 21‐1; we therefore investigated the relationship between CYFRA 21‐1 and K19^+^ HCC‐CSCs in this study.

Notably, this study demonstrates the relevance of K19 expression to preoperative serum CYFRA 21‐1 levels. Our analysis of 147 human HCC samples demonstrated that patients with K19^+^ tumors exhibited significantly higher serum CYFRA 21‐1 levels than those with K19^−^ tumors. Moreover, it should be noted that among various preoperative laboratory test values including AFP and PIVKA‐II, serum CYFRA 21‐1 level was the only statistically significant and the most sensitive predictor of K19 expression in HCC. Recently, serum *Wisteria Floribunda* agglutinin‐positive sialylated mucin 1 was reported as a marker of progenitor/biliary features in HCC 32. Further studies focusing on the appropriate combination of serum markers would enable to identify K19^+^ HCC with higher accuracy. As for the relationship between serum CYFRA 21‐1 level and patient survival, although HCC patients with high serum CYFRA levels (≥2.7 ng/mL) showed significantly shorter RFS/OS in univariate analysis, the multivariate analysis resulted that high serum CYFRA levels was not an independent poor prognostic factor in the analysis with or without K19 expression. Nevertheless, considering the significance of K19 expression as an independent poor prognostic factor both in RFS/OS and the importance of K19^+^ HCC‐CSCs for clinical application, the prediction of K19 expression by serum CYFRA 21‐1 levels is of obvious importance.

In clinical settings, imaging methods such as CT and MRI are routinely used for the diagnosis and monitoring of HCC. Additionally, we previously reported that positron emission tomography (PET) with ^18^F‐fluorodeoxyglucose (^18^F‐FDG) is useful for predicting postoperative outcomes in HCC 33, 34, and that ^18^F‐FDGPET is an effective method for identifying K19 expression in HCC tissues 22. On the other hand, serum CYFRA 21‐1 assessments are less invasive and applicable for almost all patients by peripheral blood tests, facilitating the screening of K19 expression in HCC tumors. Additionally, combining serum CYFRA 21‐1 levels with ^18^F‐FDGPET might achieve a more precise prediction of K19 expression in HCC.

Considering the dominant regulation of various signaling pathways in the maintenance of embryonic stem/progenitor cells, including the Notch, Wnt/beta‐catenin, and TGFβ/Smad signaling pathways, it is reasonable to speculate that these pathways also function in CSCs 35, 36, 37. Indeed, our previous study showed that TGFβ/Smad signaling is constitutively active in K19^+^ HCC‐CSCs, that siRNA‐based K19 knockdown suppresses pSmad2 expression in K19^+^ cells, that K19 overexpression rescues pSmad2 expression in K19^−^ cells, and that K19 is functionally associated with cell proliferation and EMT through TGFβ/Smad signaling 14, 22. These findings indicate that K19 functions as a regulator of K19^+^ HCC‐CSCs and highlight the need for further investigation into the functional relationship between K19^+^ HCC‐CSCs and CYFRA 21‐1. In this study, we used K19 promoter‐driven EGFP‐labeled cells to isolate K19^+^ populations of human HCC cell lines. Our analyses demonstrated that K19^+^ cells exhibited significantly higher CYFRA 21‐1 levels in culture supernatants. Additionally, our gain/loss of K19 function experiments clearly showed that K19 regulates supernatant levels of CYFRA 21‐1. Moreover, we showed the possibility of CYFRA 21‐1 for the treatment targeting K19^+^ HCC‐CSCs. Our previous study showed that a TGFβ receptor 1 inhibitor LY2157299 would be useful for the treatment of K19^+^ HCC in vitro and in vivo, and that TGFβ receptor 1 expression is significantly correlated with K19 expression in human HCC surgical specimens 14. In this study, we revealed that CYFRA levels of culture supernatants were significantly suppressed in K19^+^ cells treated with LY2157299, suggesting the potential clinical application for monitoring the K19 functions and the response of this compound.

Regarding the significance of K19 in HCC, the highly malignant properties of K19^+^ HCCs have been demonstrated in various studies, encouraging the development of new therapeutic strategies for K19^+^ HCC. Together with the demonstrated efficacy of TGFβ receptor 1 inhibitor against K19^+^ HCC‐CSCs, the detection of K19 expression using serum CYFRA 21‐1 before HCC treatment could allow for the actualization of individualized medicine for K19^+^ HCC patients. Further prospective clinical trials examining the usefulness of CYFRA 21‐1 as an effective method for identifying K19^+^ HCC patients and for assessing the therapeutic efficacy of TGFβ receptor 1 inhibitors in K19^+^ HCC patients will advance the clinical application of K19^+^ HCC‐CSCs.

In conclusion, the results of this study indicate that K19 regulates CYFRA 21‐1 levels in HCC cells and that CYFRA 21‐1 is an effective biomarker for identifying K19 expression in HCC. We believe that further study of K19^+^ HCC‐CSCs and CYFRA 21‐1 will contribute to the development of novel approaches for the treatment of HCC.

## Conflict of Interest

None declared.

## Supporting information


**Figure S1**. Gain/loss of K19 function in HCC cells.

**Figure S2**. EGFP labeling of the K19^+^ cells in human HCC cell lines.

**Table S1**. Clinical pathological findings of HCC patients.

**Table S2**. Primer sequences for RT‐PCR and qRT‐PCR.

**Table S3**. Univariate analysis with respect to outcome.
