# Supplementary figures and images for: Identification of keratin 19‐positive cancer stem cells associating human hepatocellular carcinoma using CYFRA 21‐1
Source: Cancer Med. 2017 Sep 30;6(11):2531–40. doi: 10.1002/cam4.1211 (PMC5673926; doi:10.1002/cam4.1211)

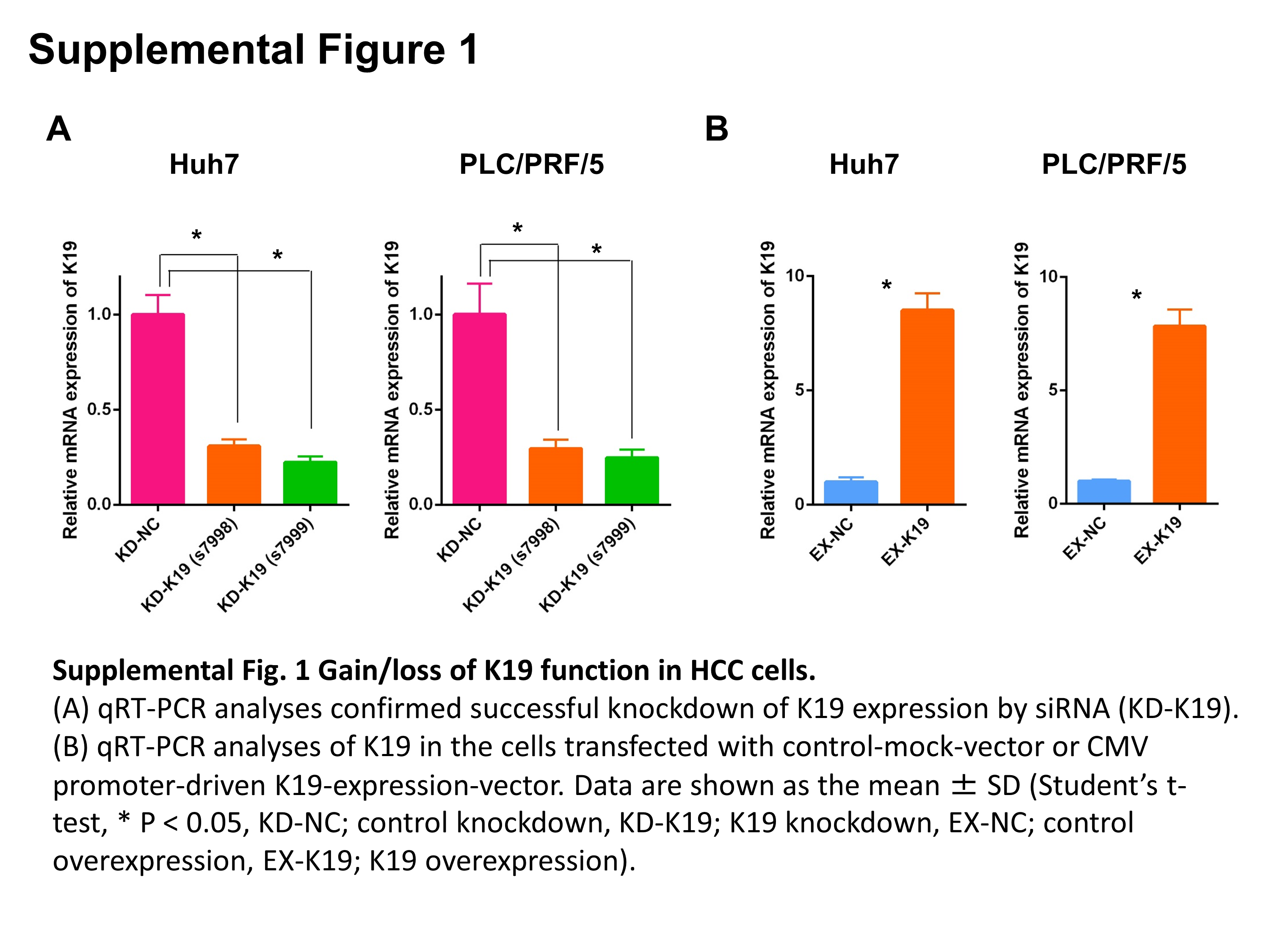

Supplement: Supplementary file 1 — Figure S1. Gain/loss of K19 function in HCC cells. [file CAM4-6-2531-s001.tif]

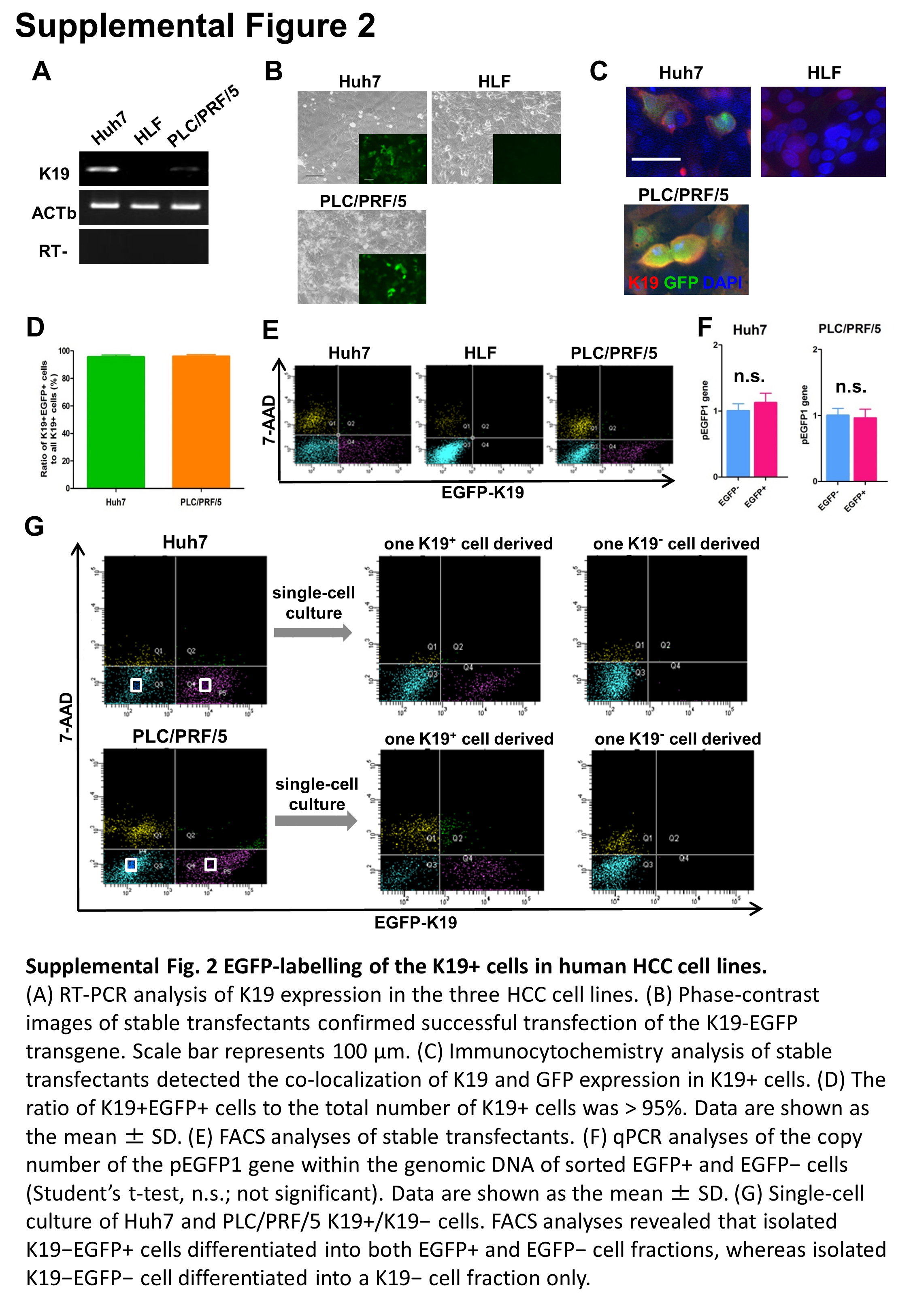

Supplement: Supplementary file 2 — Figure S2. EGFP labeling of the K19+ cells in human HCC cell lines. [file CAM4-6-2531-s002.tif]
