# Supplementary material for: Identification of keratin 19‐positive cancer stem cells associating human hepatocellular carcinoma using CYFRA 21‐1
Source: Cancer Med. 2017 Sep 30;6(11):2531–40. doi: 10.1002/cam4.1211 (PMC5673926; doi:10.1002/cam4.1211)
Supplement: Supplementary file 3 — Table S1. Clinical pathological findings of HCC patients. [file CAM4-6-2531-s003.docx]

**Supplemental Table 1**

**Clinic-pathological findings of HCC patients**

|  | K19 expression | | |
| --- | --- | --- | --- |
|  | Positive 　　　 (n=18) | Negative (n=129) | *P* value |
| Age (years, mean ± SD) | 70.2 ± 12.5 | 67.2 ± 9.9 | 0.126 |
| Gender (male/female) | 11/7 | 104/25 | 0.072 |
| Total bilirubin (> 1.0 mg/dl) | 6 | 26 | 0.226 |
| Albumin (< 3.5 g/dl) | 3 | 12 | 0.381 |
| Platelet (< 10.0×10^4^/μl) | 3 | 18 | 0.724 |
| AFP (> 20 ng/ml) | 12 | 57 | 0.083 |
| PIVKA-II (> 40 mAU/ml) | 17 | 99 | 0.122 |
| CEA (> 5.0 ng/ml) | 3 | 11 | 0.698 |
| CA 19-9 (> 37.0 U/ml) | 6 | 26 | 0.568 |
| HBs Ag positive | 3 | 28 | 0.765 |
| HCV Ab positive | 10 | 47 | 0.196 |
| Tumor size (≥ 5 cm) | 15 | 48 | < 0.001 |
| Tumor number (multiple) | 7 | 36 | 0.408 |
| Tumor differentiation |  |  | 0.035 |
| Well | 0 | 12 |  |
| Moderate | 10 | 87 |  |
| Poorly | 8 | 21 |  |
| Unknown | 0 | 8 |  |
| UICC Stage (III-IV/I-II) | 11/6 | 55/70 | 0.126 |
| Microvascular invasion | 11 | 47 | 0.069 |
| Liver cirrhosis | 3 | 40 | 0.540 |
| Type of operation |  |  | 0.117 |
| Anatomic resection | 15 | 82 |  |
| Limited resection | 3 | 47 |  |

Abbreviation: AFP, alpha-fetoprotein; PIVKA-II, protein induced by vitamin K absence or antagonists-II; CEA, carcinoembryonic antigen; CA 19-9, carbohydrate antigen 19-9; HBs Ag, hepatitis B antigen; HCV Ab, hepatitis C antibody; UICC, union for international cancer control.
