# Supplementary material for: Identification of keratin 19‐positive cancer stem cells associating human hepatocellular carcinoma using CYFRA 21‐1
Source: Cancer Med. 2017 Sep 30;6(11):2531–40. doi: 10.1002/cam4.1211 (PMC5673926; doi:10.1002/cam4.1211)
Supplement: Supplementary file 4 — Table S2. Primer sequences for RT‐PCR and qRT‐PCR. [file CAM4-6-2531-s004.docx]

**Supplemental Table 2**

**Primer sequences for RT-PCR and qRT-PCR**

| Gene | Sense primer | Antisense primer |
| --- | --- | --- |
| K19 | TTTGAGACGGAACAGGCTCT | TCAGTAACCTCGGACCTGCT |
| pEGFP1 | GTTATTACTAGCGCTACCGGACTC | CGGCCATGATATAGACGTTG |
| K19 open reading frame | ATGACTTCCTACAGCTATCG | TCAGAGGACCTTGGAGGCAG |
| ACTb | TCATGAAGATCCTCACCGAG | TTGCCAATGGTGATGACCTG |

Abbreviation: K19, keratin 19; pEGFP1, plasmid EGFP-1; ACTb, actin-beta.
