# Supplementary material for: Identification of keratin 19‐positive cancer stem cells associating human hepatocellular carcinoma using CYFRA 21‐1
Source: Cancer Med. 2017 Sep 30;6(11):2531–40. doi: 10.1002/cam4.1211 (PMC5673926; doi:10.1002/cam4.1211)
Supplement: Supplementary file 5 — Table S3. Univariate analysis with respect to outcome. [file CAM4-6-2531-s005.docx]

**Supplemental Table 3**

**Univariate analysis with respect to outcome**

| Recurrence-free survival | | | | |
| --- | --- | --- | --- | --- |
| Factors | Number of patients | Recurrence-free  survival days  (median, 95% CI) | *P* value | Hazard Ratio (median, 95% CI) |
| K19 expression |  |  |  |  |
| Positive | 18 | 193 (83-302) | < 0.001 | 38.8 |
| Negative | 129 | 1075 (767-1383) |  | (16.0-131.3) |
| Serum CYFRA 21-1 (ng/ml) |  |  |  |  |
| ≧2.7 | 13 | 399 (NA-806) | 0.040 | 1.96 |
| ＜2.7 | 134 | 923 (603-1243) |  | (1.02-3.78) |
| Age (years) |  |  |  |  |
| ≧65 | 97 | 862 (411-1312) | 0.836 | 1.04 |
| ＜65 | 50 | 894 (515-1273) |  | (0.73-1.64) |
| Gender |  |  |  |  |
| Male | 115 | 993 (686-1300) | 0.626 | 0.89 |
| Female | 32 | 546 (352-740) |  | (0.55-1.44) |
| Total billirubin (mg/dl) |  |  |  |  |
| ＞1.0 | 32 | 665 (NA-1433) | 0.294 | 1.30 |
| ≦1.0 | 115 | 894 (510-1277) |  | (0.80-2.14) |
| Albumin (g/dl) |  |  |  |  |
| ＜3.5 | 15 | 821 (471-1171) | 0.685 | 1.15 |
| ≧3.5 | 132 | 1410 (39-2781) |  | (0.58-2.27) |
| Platelet count (10^4^/mm^3^) |  |  |  |  |
| ＜10 | 21 | 725 (NA-1630) | 0.076 | 1.79 |
| ≧10 | 126 | 1016 (578-1454) |  | (0.94-3.40) |
| AFP (ng/ml) |  |  |  |  |
| ＞20 | 69 | 544 (167-921) | 0.104 | 1.40 |
| ≦20 | 78 | 1053 (628-1478) |  | (0.93-2.08) |
| PIVKA-II (mAU/ml) |  |  |  |  |
| ＞40 | 116 | 572 (314-830) | 0.074 | 1.50 |
| ≦40 | 31 | 1410 (983-1837) |  | (0.96-2.34) |
| CEA (ng/ml) |  |  |  |  |
| ＞5.0 | 14 | 630 (NA-1262) | 0.723 | 1.14 |
| ≦5.0 | 96 | 665 (256-1074) |  | (0.56-2.30) |
| CA 19-9 (U/ml) |  |  |  |  |
| ＞37.0 | 32 | 821 (200-1442) | 0.973 | 1.01 |
| ≦37.0 | 78 | 665 (138-1192) |  | (0.61-1.66) |
| Hepatitis B infection |  |  |  |  |
| Present | 31 | 821 (454-1188) | 0.478 | 1.17 |
| Absent | 116 | 1070 (373-1767) |  | (0.72-1.89) |
| Hepatitis C infection |  |  |  |  |
| Present | 57 | 923 (244-1602) | 0.824 | 0.97 |
| Absent | 90 | 862 (426-1298) |  | (0.64-1.46) |
| Tumor size (cm) |  |  |  |  |
| ≧5cm | 63 | 490 (305-675) | 0.042 | 1.57 |
| ＜5cm | 84 | 1053 (696-1410) |  | (1.04-2.34) |
| Tumor number |  |  |  |  |
| Multiple | 43 | 452 (187-717) | 0.002 | 2.13 |
| Single | 104 | 1129 (765-1493) |  | (1.32-3.43) |
| Tumor differentiation |  |  |  |  |
| Poor | 29 | 202 (NA-507) | 0.006 | 2.33 |
| Others | 109 | 1016 (722-1310) |  | (1.27-4.28) |
| Microvascular invasion |  |  |  |  |
| Positive | 58 | 436 (184-688) | 0.024 | 1.70 |
| Negative | 89 | 1078 (827-1329) |  | (1.10-2.61) |
| Liver cirrhosis |  |  |  |  |
| F4 | 43 | 1017 (746-1288) | 0.891 | 1.02 |
| Others | 104 | 862 (384-1339) |  | (0.64-1.61) |
| Type of resection |  |  |  |  |
| Anatomic | 97 | 585 (151-1019) | 0.851 | 1.04 |
| Partial | 50 | 1016 (550-1482) |  | (0.68-1.57) |
| Overall survival | | | | |
| Factors | Number of patients | Overall  survival days (median, 95%CI) | *P* value | Hazard Ratio (median, 95% CI) |
| K19 expression |  |  |  |  |
| Positive | 18 | 546 (405-687) | < 0.001 | 25.0 |
| Negative | 129 | 2893 (2619-3167) |  | (8.76-71.5) |
| Serum CYFRA 21-1 (ng/ml) |  |  |  |  |
| ≧2.7 | 13 | 678 (NA-1680) | 0.031 | 2.15 |
| ＜2.7 | 134 | 2757 (2392-3122) |  | (1.06-4.36) |
| Age (years) |  |  |  |  |
| ≧65 | 97 | 2449 (1710-3187) | 0.097 | 1.18 |
| ＜65 | 50 | 2893 (2607-3179) |  | (0.70-2.01) |
| Gender |  |  |  |  |
| Male | 115 | 2685 (2314-3055) | 0.543 | 1.21 |
| Female | 32 | 3346 |  | (0.66-2.21) |
| Total billirubin (mg/dl) |  |  |  |  |
| ＞1.0 | 32 | 2466 (1220-3712) | 0.435 | 1.28 |
| ≦1.0 | 115 | 2757 (2374-3140) |  | (0.69-2.37) |
| Albumin (g/dl) |  |  |  |  |
| ＜3.5 | 15 | 1691 (879-2503) | 0.202 | 1.73 |
| ≧3.5 | 132 | 2757 (2385-3129) |  | (0.75-4.00) |
| Platelet count (10^4^/mm^3^) |  |  |  |  |
| ＜10 | 21 | 2466 (535-4397) | 0.245 | 1.53 |
| ≧10 | 126 | 2893 (2628-3158) |  | (0.75-3.11) |
| AFP (ng/ml) |  |  |  |  |
| ＞20 | 69 | 2173 (1372-2974) | 0.026 | 1.74 |
| ≦20 | 78 | 2893 (2624-3162) |  | (1.07-2.83) |
| PIVKA-II (mAU/ml) |  |  |  |  |
| ＞40 | 116 | 2757 (2350-3164) | 0.499 | 1.21 |
| ≦40 | 31 | 2685 (2215-3155) |  | (0.69-2.12) |
| CEA (ng/ml) |  |  |  |  |
| ＞5.0 | 14 | 1273 (712-1834) | 0.056 | 2.25 |
| ≦5.0 | 96 | 2921 (1643-4199) |  | (0.98-5.14) |
| CA 19-9 (U/ml) |  |  |  |  |
| ＞37.0 | 32 | 2449 (1363-3535) | 0.837 | 1.06 |
| ≦37.0 | 78 | 2757 (1900-3614) |  | (0.59-1.90) |
| Hepatitis B infection |  |  |  |  |
| Present | 31 | NA | 0.448 | 1.25 |
| Absent | 116 | 2685 (2063-3307) |  | (0.70-2.24) |
| Hepatitis C infection |  |  |  |  |
| Present | 57 | 2893 (2491-3295) | 0.933 | 0.98 |
| Absent | 90 | 2646 (2301-2991) |  | (0.60-1.60) |
| Tumor size (cm) |  |  |  |  |
| ≧5cm | 63 | 1832 (1402-2262) | 0.002 | 2.21 |
| ＜5cm | 84 | 2921 (2704-3138) |  | (1.33-3.65) |
| Tumor number |  |  |  |  |
| Multiple | 43 | 1659 (1197-2121) | 0.011 | 2.06 |
| Single | 104 | 2921 (2534-3308) |  | (1.18-3.58) |
| Tumor differentiation |  |  |  |  |
| Poor | 29 | 1392 (1022-1762) | < 0.001 | 3.95 |
| Others | 109 | 2893 (2637-3149) |  | (1.99-7.84) |
| Microvascular invasion |  |  |  |  |
| Positive | 58 | 2173 (1164-3182) | 0.020 | 1.84 |
| Negative | 89 | 2921 (2632-3210) |  | (1.10-3.07) |
| Liver cirrhosis |  |  |  |  |
| F4 | 43 | 2534 (2001-3067) | 0.425 | 1.25 |
| Others | 104 | 2950 (2643-3257) |  | (0.72-2.18) |
| Type of resection |  |  |  |  |
| Anatomic | 97 | 2757 (2413-3101) | 0.431 | 0.82 |
| Partial | 50 | 2685 (2287-3082) |  | (0.50-1.34) |

Abbreviation: K19, keratin 19; CYFRA 21-1, cytokeratin 19 fragment; AST, asparatate aminotransferase; ALT, alanine aminotransferase; AFP, alpha-fetoprotein; PIVKA-II, protein induced by vitamin K absence or antagonists-II; CEA, carcinoembryonic antigen; CA 19-9, carbohydrate antigen 19-9; NA, not available; CI, confidence interval.
